# MicroRNA involvement in mechanism of endogenous protection induced by fastigial nucleus stimulation based on deep sequencing and bioinformatics

**DOI:** 10.1186/s12920-015-0155-4

**Published:** 2015-11-23

**Authors:** Ling-Bo Feng, Xiao-Min Pang, Lei Zhang, Jin-Pin Li, Li-Gang Huang, Sheng-You Su, Xia Zhou, Sheng-Hua Li, Hui-Yao Xiang, Chun-Yong Chen, Jing-Li Liu

**Affiliations:** Department of Neurology, the First Affiliated Hospital, Guangxi Medical University, Nanning, China; Department of Neurology, the First People’s Hospital of Nanning, Guangxi Medical University, Nanning, China; Department of Neurology, Dongguan Kanghua hospital, Dongguan, Guangdong China

**Keywords:** miRNA, FNS, Neuroprotection, Deep sequencing, Microarray

## Abstract

**Background:**

Neurogenic neuroprotection is a promising approach for treating patients with ischemic brain lesions. Fastigial nucleus stimulation (FNS) has been shown to reduce the tissue damage resulting from focal cerebral ischemia in the earlier studies. However, the mechanisms of neuroprotection induced by FNS remain unclear. MicroRNAs (miRNAs) are a newly discovered group of non-coding small RNA molecules that negatively regulate target gene expression and involved in the regulation of pathological process. To date, there is a lack of knowledge on the expression of miRNA in response to FNS. Thus, we study the regulation of miRNAs in the rat ischemic brain by the neuroprotection effect of FNS.

**Methods:**

In this study, we used an established focal cerebral ischemia/reperfusion (IR) model in rats. MiRNA expression profile of rat ischemic cortex after 1 h of FNS were investigated using deep sequencing. Microarray was performed to study the expression pattern of miRNAs. Functional annotation on the miRNA was carried out by bioinformatics analysis.

**Results:**

Two thousand four hundred ninety three miRNAs were detected and found to be miRNAs or miRNA candidates using deep sequencing technology. We found that the FNS-related miRNAs were differentially expressed according microarray data. Bioinformatics analysis indicated that several differentially expressed miRNAs might be a central node of neuroprotection-associated genetic networks and contribute to neuroprotection induced by FNS.

**Conclusions:**

MiRNA acts as a novel regulator and contributes to FNS-induced neuroprotection. Our study provides a better understanding of neuroprotection induced by FNS.

**Electronic supplementary material:**

The online version of this article (doi:10.1186/s12920-015-0155-4) contains supplementary material, which is available to authorized users.

## Background

Ischemic stroke is one of the leading causes of death and disabilities globally and the options of therapeutic intervention remained remarkably limited [1–3]. Fastigial nucleus stimulation (FNS) provides long-lasting protection against ischemic infarction, which is emerging as one of promising approaches for treating patients with ischemic brain lesions [4]. The fastigial nucleus (FN) is located at the top of the fourth ventricle and includes adrenergic intrinsic neurons and nerve fibers [5]. Fibers passing through the FN can be excited by electrical stimulation, leading to a series of reactions including reflexive vascular expansion, elevated blood pressure and increased cerebral blood flow [6, 7]. Its biological function is far more complicated than what we know. Based on the previous study results made by us and other researchers, a 1-h electrical stimulation of the FN reduces the volume of focal ischemic infarction produced by occluding the middle cerebral artery (MCAO) in rats [4, 8]. The area of salvage is confined to the ischemic penumbra. This protection persisted for 10 but disappeared by 30 d [6, 9]. Important data in the last two decades have demonstrated that the neuroprotection initiated by stimulation of FN is involving multiple mechanisms, including the inhibition of inflammatory response, excitotoxic damage as well as apoptosis [10–13]. Although the effects of experimental FNS have been well established, the mechanisms underlying this neurogenic neuroprotection remain a topic of intense investigation.

MicroRNAs (miRNAs), a large family of ~22 nucleotide (nt) single-stranded non-coding small RNAs, are derived from ~70 nt long stem-loop precursors (pre-miRNAs) [14, 15]. They are found in various species of animals, plants and viruses. miRNAs regulate the expression of target gene by promoting mRNA degradation or inhibiting translation at the post-transcriptional [16]. Specific miRNAs have been reported to play important roles in a wide range of physiological and pathological processes [17–20]. Recently, studies have suggested that miRNA may be an inducible factor to response on the neuroprotection of ischemic pre-conditioning (IPC) through their function in regulating gene expression [21, 22]. To date, however, whether FN stimulation exerts its effect through miRNAs regulation mechanisms remains unclear.

Thus, in this study, we investigated the miRNA profiles after FNS and associated neuroprotective mechanisms for the first time. Our study would provide a basis for further unravelling the mechanism on how miRNAs directly responsible for FN stimulation, and its relevance to diverse biological processes of neuroprotection induced by FNS.

## Methods

### Animals

Male Sprague-Dawley rats (280–300 g) were purchased from the Animal Experiment Center of Guangxi Medical University. Animals were handled according to the guidelines of the Council for International Organization of Medical Sciences on Animal Experimentation (World Health Organization, Geneva, Switzerland). The Guangxi Medical University Animal Care and Use Committee approved the animal protocols.

### Electrical stimulation of the FN and transient focal cerebral ischemia

Rats were anesthetized with an intraperitoneal injection of 3.5 % chloral hydrate (1.0 ml/100 g, Sigma, Missouri, USA). All efforts were made to minimize suffering. The fastigial nucleus was accurately targeted using a stereotaxic atlas of the rat brain. The posterior border of bregma was set as the zero point, and a hole (1.1 mm lateral, 11.1 mm posterior, 5.6 mm deep) was made for electrode attachment. An YC-2 programmed electrical stimulation instrument (Chengdu Instrument Factory, Chengdu, China) was used to apply a 70-μA direct-current square-wave pulse (50 Hz). Each electrical stimulation lasted 1 h.

An established focal cerebral ischemia/reperfusion (I/R) model was employed 24 h later. Focal cerebral ischemia was induced in rats with the transient middle cerebral artery occlusion (MCAO) as previously described [23]. A 0.285 mm diameter nylon monofilament suture was used and the occlusion was maintained for 2 h. Rats in the sham-operated group underwent only vascular separation without filament insertion. Thereafter, the reperfusion was performed for various times (3 h, 6 h, 12 h, 24 h or 72 h).

### RNA isolation

After 1 h of FNS in rats subjected to 2 h of MCAO and various times (3 h, 6 h, 12 h, 24 h or 72 h) of reperfusion, brain tissue sections (approximately 2 mm thick) from the point behind the optic chiasm in the ipsilateral cortex were quickly dissected and harvested on ice. Total RNA was extracted using Trizol reagent (Invitrogen, California, USA) following the manufacturer’s procedure. The quantity and purity of total RNA were monitored via analysis by NanoDrop ND-2000 spectrophotometer (Nano Drop, Delaware, USA) at 260/280 nm (ratio 1.8–2.0). The integrity was analyzed using a Bioanalyzer 2100 and RNA 6000 Nano Lab Chip Kit (Agilent, California, USA) with RIN number >7.0.

### Small RNA library preparation and sequencing

Approximately 1 μg of total RNA were used to prepare small RNA library according to protocol of TruSeq Small RNA Sample Prep Kit (Illumina, San Diego, USA). Briefly, RNA molecules were ligated to 5’ and 3’ adaptors sequentially and converted to cDNA by reverse transcription followed by PCR amplification. The amplification products (around 140 bp) were excised from a 6 % polyacrylamide Tri-borate-EDTA gel. The purified cDNA library was used for cluster generation on Illumina’s Cluster Station, and single-end sequencing (36 bp) was performed on an Illumina Hiseq 2500 at the LC-BIO (Hangzhou, China) following the manufacturer’s instructions for instrument use. Raw sequencing reads were obtained using Illumina’s Sequencing Control Studio software version 2.8 (SCS v2.8) following real-time sequencing image analysis and base-calling by Illumina’s Real-Time Analysis version 1.8.70 (RTAv1.8.70).

### Standard small RNA analysis

A proprietary pipelinescript, ACGT101-miR v4.2 (LC Sciences, Houston, Texas, USA), was used for sequencing data analysis. Following the removal of adapter sequences, low-quality reads and common RNA families (rRNA, tRNA, snRNA, snoRNA), unique sequences of 18–26 bases were mapped to *Rattus norvegicus* precursors in miRBase 20.0 using a BLAST search to identify known miRNAs and novel 3p- and 5p- derived miRNAs. The unique sequences mapping to *Rattus norvegicus* mature miRNAs in hairpin arms were identified as known miRNAs. The unique sequences mapping to the other arm of known *Rattus norvegicus* precursor hairpin opposite to the annotated mature miRNA-containing arm were considered to be novel 5p- or 3p- derived miRNA candidates. The remaining sequences were mapped to other mammals’ precursors in miRBase using further BLAST searches. The unmapped sequences were analyzed to predict potential novel miRNAs. A BLAST search was performed against the *Rattus norvegicus* genome, and the sequences that contain hairpin RNA structures were predicted from the flanking 80-nt sequences using RNAfold software (http://rna.tbi.univie.ac.at/cgi-bin/RNAfold.cgi).

### Microarray analysis

The miRNA microarray assays were performed by a service provider (LC Sciences, Houston, USA). The customμparaflo™ microfluidic chip contained 728 unique miRNAs listed in Sanger miRBase release 20.0 and 479 predicted miRNA sequences detected from our sequencing results. 5 μg total RNA was fractioned with a YM-100 Microcon centrifugal filter (Millipore, Billerica, USA) and the mall RNAs (<300 nt) isolated were 3’-extended with a poly (A) tail by poly (A) polymeras. Anoligonucleotide tag was then ligated to the poly (A) tail for laterfluorescent dye staining. Hybridization was performed on a μParaflo™ microfluidic chip with 100 μl 6 × SSPE buffer containing 25 % formamide at 34 °C overnight using a micro-circulation pump (Atactic Technologies, Houston, TX, USA). After hybridization, fluorescence labeling using tag-specific Cy5 dyes was used for detection. Hybridization images were collected by using a laser scanner (GenePix 4000B, Molecular Device, Sunnyvale, CA, USA) and digitized by Array-Pro image analysis software (Media Cybernetics, Bethesda, Maryland, USA). Data were analyzed by subtracting the background and then normalizing the signals using a LOWESS filter (Locally-weighted Regression). For two-color experiments, the ratio of the two sets of detected signals (log2-transformed and balanced) and *P*-values of the *t*-test were calculated; a *P*-value <0.01 was considered as differentially expressed.

### Target prediction and gene ontology analysis

For miRNAs expressed in significant amounts, we used the miRanda algorithm base (http://www.microrna.org/microrna/home.do) and TargetScan (http://www.targetscan.org/) to predict miRNA target genes. Finally, the data predicted by both algorithms were combined and the overlaps were calculated. The most abundant miRNAs targets were also annotated using Gene Ontology (GO) categories (http://www.geneontology.org/) and the Kyoto Encyclopedia of Genes and Genomes (KEGG) (http://www.genome.jp/kegg/pathway.html).

## Results

### Analysis and annotation of sequencing data

**Fig. 1:**
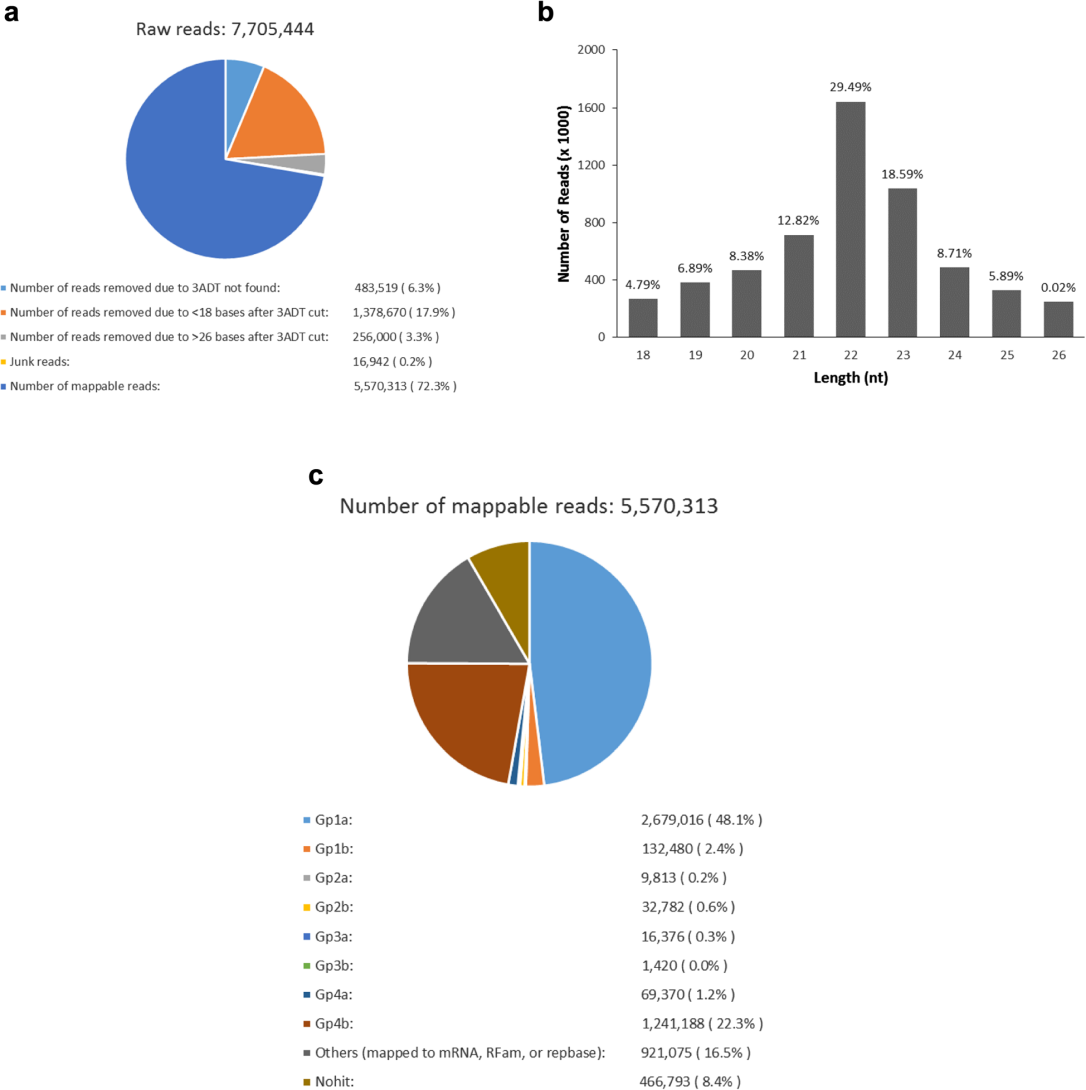
Generation and analysis of sequenced small RNAs from rat ischemic cortex tissue after FN stimulation. **a** Distribution of 7,705,444 raw sequences obtained using Solexa deep sequencing. **b** Length distribution of sequenced small RNA. **c** Categorical distribution of 5,570,313 mappable reads

A cDNA library of small RNAs was prepared from a mixture of rat ischemic cortex tissue at 5 time points (3 h, 6 h, 12 h, 24 h and 72 h) of reperfusion after 1 h of FNS. A total of 7,705,444 raw sequences of small RNAs were obtained using Solexa deep sequencing. Adapter sequences, junk and low-quality reads were removed, and 5,570,313 clean reads (72.3 %) of 18 and 26 bases in length were processed for sequencing analysis (Fig. 1a). Of these reads, the majority of the small RNAs were 20–24 nt in size, with the predominant species being 22 nt in length, which is typical size range for Dicer-processed products (Fig. 1b).

To identify the known miRNAs in rat after FNS, the sequencing data were processed through Illumina’s Genome Analyzer Pipeline and the ACGT-miR program (Additional file 1: Figure S1). Certain RNA reference sequences including mRNA, rRNA, tRNA, snRNA, snoRNA and Rfam (921,075 reads, 16.5 %) were filtered from the mappable reads. The remaining 4,649,238 reads were subjected to advance bioinformatics analysis (Fig. 1c).

Among the clean reads, total 2,679,016 reads (Group 1 a) that represent 620 unique sequences were mapped to known *Rattus norvegicus* pre-miRNAs and mature miRNAs. In addition, 132,480 reads (Group 1b) represent 112 unique sequences mapped to other known mammalian pre-miRNAs and miRNAs, but novel to *Rattus norvegicus*. The above two group we identified as known miRNAs.

According to the standard criteria for novel miRNA identification, extended sequences at the aligned genome locations have a propensity to form hairpin structures. 9813 reads (Group 2a) represent 42 unique sequences mapped to known pre-miRNAs and genome; with the extended sequences at the mapped positions of the genome potentially form hairpins. They are considered to be novel 5p- or 3p- derived miRNA candidates. 69,370 reads (Group 4a) represent 781 unique sequences unmapped to known miRNAs but mapped to genome and the hairpin RNA structures containing sequences were predicated from the flank 80 nt sequences using RNAfold software. This part is identified as novel miRNAs in rat. Known and predicted miRNAs are summarized in Table 1.

**Table 1:** Known and predicted miRNAs

	Group	Unique miRs
Known miRs		
Of specific species	Group 1a	620
Of selected species, but novel to specific species	Group 1b	112
Predicted miRs		
Mapped to known pre-miRs of selected species and genome; within hairpins	Group 2a	42
Mapped to known pre-miRs of selected species and genome; no hairpins	Group 2b	374
Mapped to known pre-miRs and miRs of selected species but unmapped to genome	Group 3a	197
Mapped to known pre-miRs of selected species but unmapped to genome	Group 3b	561
Unmapped to known miRs but mapped to genome and within hairpins	Group 4a	781
Overall (Unique miRNAs)		2493

Specific species: Rattus norvegicus; Specific species: Mammalia

### The most abundantly expressed known miRNAs

In high-throughput sequencing, the expression level of a unique miRNA can be measured by the frequency of its read count. Among the 2493 unique miRNAs, the frequency of various miRNAs varied drastically, with high-copy (counts > 10,000) of the frequent sequences taking up 22, whereas 1791 miRNAs were sequenced with five or fewer read counts. To assess the expression of known miRNAs after 1 h of FNS in focal ischemic rat brain, we analyzed all 620 mappable known miRNAs in the dataset. Their counts ranged from 1 to 94,525 sequence reads. The top 20 of most highly expressed known miRNAs are listed in Table 2.

**Table 2:** Top 20 of the most abundantly expressed miRNAs

miR_name	miR_seq	len	Copy of the miRNA
rno-miR-125b–5p	TCCCTGAGACCCTAACTTGTGA	22	94,525
rno–miR–219a–2–3p	AGAATTGTGGCTGGACATCTGT	22	92,142
rno–miR–127–3p	TCGGATCCGTCTGAGCTTGGCT	22	28,705
rno–let–7f–5p	TGAGGTAGTAGATTGTATAGTT	22	18,363
rno–let–7i–5p	TGAGGTAGTAGTTTGTGCTGTT	22	13,509
rno–miR–27b–3p	TTCACAGTGGCTAAGTTCTGC	21	10,614
rno–let–7c–5p	TGAGGTAGTAGGTTGTATGGTT	22	10,424
rno–miR–128–3p	TCACAGTGAACCGGTCTCTTT	21	8521
rno–miR–379–5p	TGGTAGACTATGGAACGTAGG	21	8379
rno–miR–181a–5p	AACATTCAACGCTGTCGGTGAGT	23	8345
rno–miR–132–3p	TAACAGTCTACAGCCATGGTCG	22	7192
rno–miR–29b–3p	TAGCACCATTTGAAATCAGTGTT	23	6772
rno–let–7a–5p	TGAGGTAGTAGGTTGTATAGTT	22	6511
rno–let–7b–5p	TGAGGTAGTAGGTTGTGTGGTT	22	6388
rno–miR–218a–5p	TTGTGCTTGATCTAACCATGT	21	6104
rno–miR–129–5p	CTTTTTGCGGTCTGGGCTTGC	21	5216
rno–miR–434–3p	TTTGAACCATCACTCGACTCCT	22	5113
rno–miR–99b–5p	CACCCGTAGAACCGACCTTGCG	22	4921
rno–miR–25–3p	CATTGCACTTGTCTCGGTCTGA	22	3456
rno–miR–148b–3p	TCAGTGCATCACAGAACTTTGT	22	3332

MiRNAs regulate expression of specific gene via hybridization to mRNA transcripts to promote RNA degradation, inhibit translation or both. To better understand the biological functions of the most abundant miRNAs involved in FNS, putative target genes were predicted using by targetscan and miranda. The target genes of top 20 abundant miRNAs were predicted. In total, 14,947 annotated mRNA transcripts were predicted. To further understand the biological significance of this abundant miRNAs, predicted targets of GO terms and KEGG pathway were introduced. The putative target genes of abundant miRNAs appeared to be involved in a broad range of biological processes. Many signaling pathways were found to be involved, including Metabolic pathway, MAPK signaling pathway, Calcium signaling pathway, Neurotrophin signaling pathway, VEGF signaling pathway and apoptosis (Additional file 2: Table S1).

### MiRNA expression patterns after FNS in the ischemic cortex

**Fig. 2:**
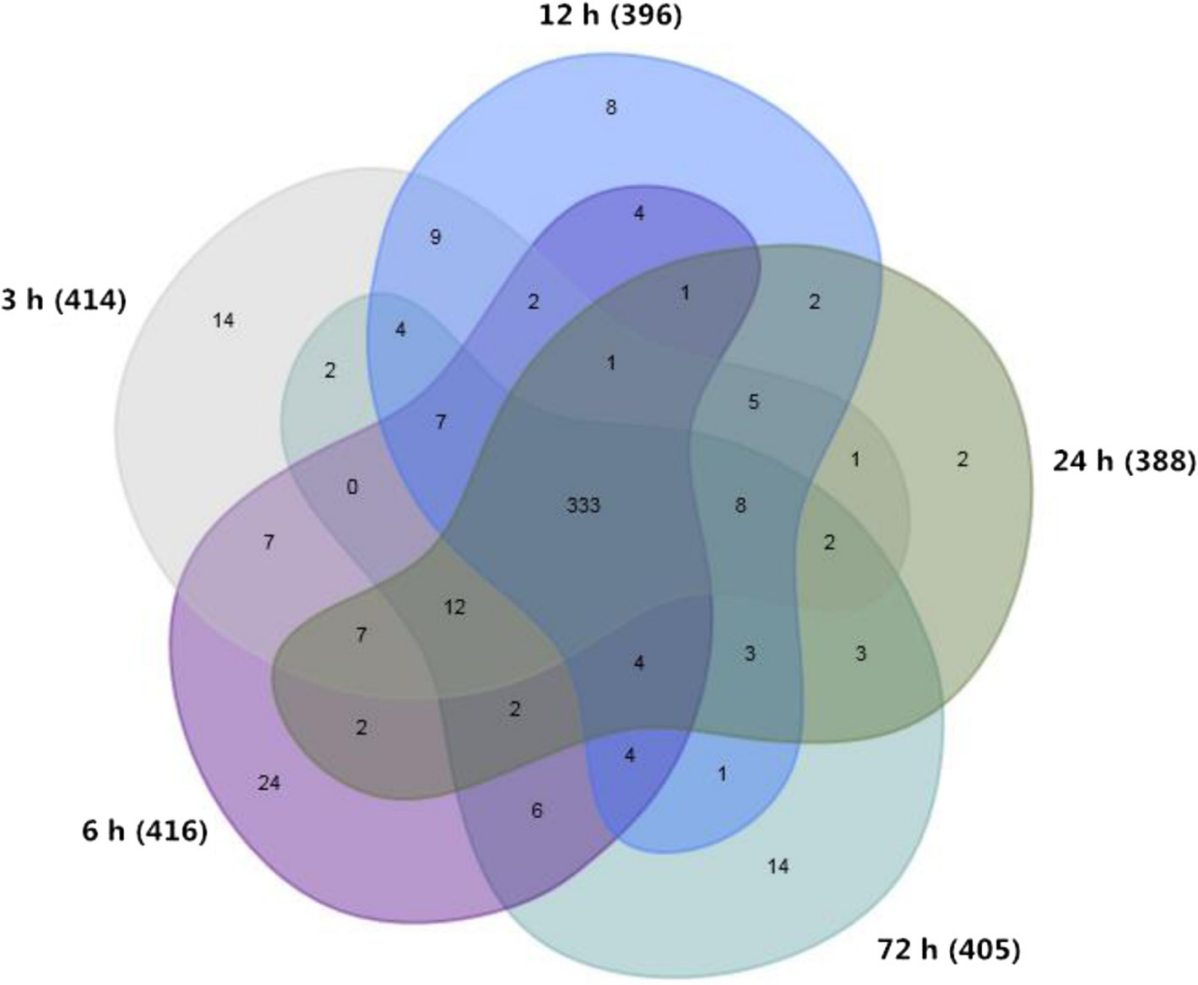
Differentially expressed miRNAs among five groups of different reperfusion time piont. Numbers presented the number of miRNAs expressed in a group or overlapping between groups

Given the low percentage of counts for the low-copy groups and the need for replicates due to their higher possibility of sequencing errors, we designed a specific custom miRNA microarray chip excluding low-copy sequences to analyze the miRNAs expression profiles. Following treatment with 1 h FNS or non-FNS, rats were subjected to 2 h MCAO and sacrificed at 3 h, 6 h, 12 h, 24 h or 72 h of reperfusion respectively. RNA from the ischemic cortex was collected to performed microarray analysis. 505 detectable miRNA transcripts in the miRNA microarrays expressed at various time point, of which 414 miRNAs were detected at 3 h, 416 miRNAs were detected at 6 h, 396 miRNAs were detected at 12 h, 388 miRNAs were detected at 24 h, 405 miRNAs were detected at 72 h, and only 333 miRNAs were detected in all groups (Fig. 2).

**Fig. 3:**
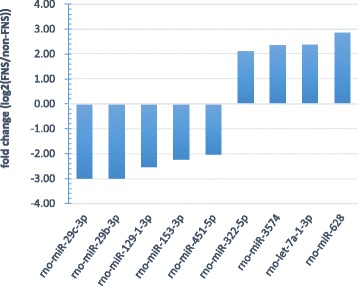
Differentially expressed miRNAs at 12 h of reperfusion after FNS. *P* < 0.01

**Fig. 4:**
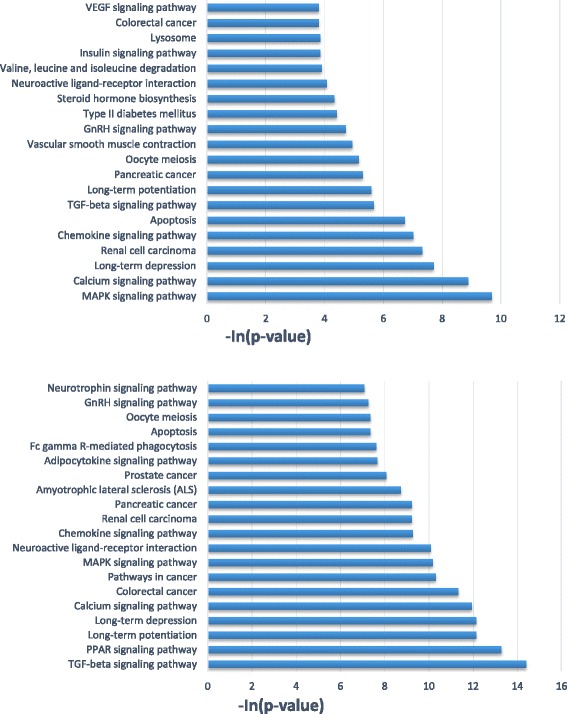
The top KEGG pathways of biological function of the targets of 9 differentially expressed miRNAs altered after FNS. The top 20 KEGG pathways targeted by the up-regulated and the downregulated miRNAs were shown in the top and bottom panels, respectively. The vertical axis was the pathway category and the horizontal axis was the –In(*p*-value) of pathway

The expression profile of miRNAs provides a hint of their potential functions. At 12 h time points, a total of 24 miRNAs showed differential expression with ∣log2∣ > 2, which was the most among the all of time points (Additional file 3: Table S2). Of these miRNAs, rno-miR-129-1-3p, rno-miR-153-3p, rno-miR-29b-3p, rno-miR-29c-3p and rno-miR-451-5p were down-regulated, whereas rno-let-7a-1-3p, rno-miR-322-5p, rno-miR-3574 and rno-miR-628 were observed to be highly upregulated with *p* < 0.01 (Fig. 3). These differentially expressed miRNAs may play an important role in the protection after FNS. Accordingly, target prediction of these 9 differentially expressed miRNAs were performed. The target genes predicted to be related to Calcium signaling pathway, PPAR signaling pathway, TGF-beta signaling pathway, Neurotrophin signaling pathway, MAPK signaling pathway and so on (Fig. 4).

## Discussion

FNS may offer a new approach to neuroprotection against cerebral ischemia. FNS has been showed to promote nerve tissue repair, reconstruction, neurological rehabilitation and improve stroke-related complication [9, 10]. Nevertheless, the molecular mechanisms that regulate gene expression during cerebral ischemia after FNS are still not completely understood. Recently, increasing attention has been focused on miRNAs, which are promising targets in the molecular mechanisms of gene regulation, pathology, and drug actions [24, 25]. To date, the neural functional relevance of such a central neural neuroprotective network induced by FNS have yet to be revealed. Our study presented the first profile of miRNA expression in the ischemic cortex after 1 h of FNS. The differentially expressed miRNAs may participate in neuroprotection induced by FNS, and provides further insights into miRNAs functions in ischemic injury.

In the present study, we used Solexa sequencing technology on small RNA libraries prepared from a mixture sample of rat ischemic cortex at 5 time points (3, 6, 12, 24 and 72 h) of reperfusion after FNS. Total, 7,705,444 reads were detected, out of which 5,570,313 were clean reads and represents 2493 unique tags. In all, 75.1 % of the mappable sequences were found to be miRNAs or miRNA candidates. High throughput sequencing not only has the ability to identify miRNAs, and to some extent reflects the gene expression information. The copy numbers of miRNAs sequence in frequency response the relative abundance of its expression. Among the sequenced sequences, the copy numbers of 117 miRNAs greater than 10,000. We found that the most abundantly expressed miRNAs are miR-125b-5p. miR-125b has been described to be involved in different cellular processes such as inflammation, cell proliferation, and cell cycle regulation [26–29]. Other miRNAs that were abundantly expressed after FNS include the *let-7* family members. *Let-7* and its family members are highly conserved across species in sequence [30, 31]. Despite their high level of expression in the brain, the function in the central nervous system is poorly addressed. The research about these miRNAs will deepen our understanding of the pathogenesis of neuroprotection induced by FNS.

To date, a total of 728 rat mature miRNAs were included in the miRBase database (release 20) and many rat miRNAs remain to be discovered. Given the importance of rat as a model organism, discovery of the completed set of rat miRNAs is necessary for understanding rat miRNA regulation. The identification of FNS-related miRNAs by our deep sequencing analysis shows that the dataset is reliable not only for characterizing expression profiles of known miRNAs but also for discovery of novel miRNAs. However, we are not able to determine how many miRNAs would be missing in our study, because the sample for the high-through sequencing was the mixed ischemic cortex. We discovered a lot of potential novel miRNAs, such as PC-5p-603_1535, PC-3p-3469_406, PC-5p-3581_373, PC-5p-9723_139, ect. The identification of novel miRNA has been expounded in another article [32]. In this paper, we do not repeat narrative again.

Microarray data of miRNAs after FNS treatment in ischemic rat cortex revealed significant changes in several miRNAs. The differential expression profile of miRNAs provides a hint of their potential functions during neuroprotection. 9 miRNAs (rno-miR-129-1-3p, rno-miR-153-3p, rno-miR-29b-3p, rno-miR-29c-3p, rno-miR-451-5p, rno-let-7a-1-3p, rno-miR-322-5p, rno-miR-3574 and rno-miR-628) showed statistically significant change. MiRNAs are a class of sophisticated gene expression regulators that inhibit translation and/or degrade target mRNAs by recognizing them through base pairing with short regions near 3’-UTRs. Hence, miRNAs can target various mRNAs that could be part of a biological pathway. The putative target genes of differentially expressed miRNAs appeared to be involved in Calcium signaling pathway, PPAR signaling pathway, TGF-beta signaling pathway, Neurotrophin signaling pathway, MAPK signaling pathway and so on. PPARs act as nuclear receptor and its activation induces a decrease in neuronal death by prevention of oxidative or inflammatory mechanisms implicated in cerebral injury [33]. Neurotrophins are a family of trophic factors involved in differentiation and survival of neural cells, which have now been shown to mediate both positive and negative survival signals, by signalling through the Trk and p75 neurotrophin receptors, respectively [34, 35]. TGF-βs regulate a wide spectrum of cellular functions such as proliferation, apoptosis, differentiation and migration. TGF-beta signaling is a molecular mechanism which limit neuroinflammation, and activate TGF-beta in the peri-infarct cortex and preserve brain function during the subacute period after stroke [11, 36]. The MAP-kinase family members are known to be stimulated after cerebral ischemia and were thought to regulate signal transduction, gene expression and metabolism. Based on the above information, we hypothesize that these differentially expressed miRNAs serve as mediators of the brain’s response to FNS that leads to endogenous neuroprotection. On the other hand, our research investigated the role of miR-29c-3p in the neuroprotection induced by FNS after ischemic injury and we found that miR-29c-3p attenuated ischemic neuronal death by negatively regulating apoptotic proteins Birc2 and Bak1 associated with the PI3K-Akt signaling pathway [37]. Our finding expand the understanding of miRNAs associated with ischemic cerebral disease and may provide a basis for novel therapeutic strategies aimed at enhancing tissue and cell survival in the ischemic stroke.

## Conclusion

In conclusion, our study presented the first profile of miRNA expression in the ischemic cortex after 1 h of FNS. Several miRNAs exhibited significantly differential expression. Bioinformatics analysis indicated that these differentially expressed miRNAs may target various mRNAs involved in biological pathway and act as endogenous neuroprotection effectors. Next, we would focus on elucidating the interactions and regulatory networks of specific miRNAs, which leading to endogenous neuroprotection by in vivo experiments.

### Availability of supporting information

The Solexa sequencing data supporting the results of this article are available in the LabArchives repository (https://mynotebook.labarchives.com/share/FNS%2520sequencing/MjAuOHwxMzcyODYvMTYvVHJlZU5vZGUvMTkxMDQzMTcwNXw1Mi44).

## Additional files

Additional file 1: Figure S1. Showed the analysis workflow of the deep sequencing. (PDF 97 kb) Additional file 2: Table S1. Showed the enriched GO terms and KEGG pathway of predicted targets of top 20 abundant miRNAs. (PDF 15 kb) Additional file 3: Table S2. Showed the differentially expression pattern after 1-h of FNS. (PDF 321 kb)

## Abbreviations

FNS: Fastigial nucleus stimulation
miRNA: microRNA
FN: Fastigial nucleus
I/R: Ischemia/reperfusion
MCAO: Middle cerebral artery occlusion
GO: Gene Ontology
KEGG: Kyoto Encyclopedia of Genes and Genomes
UTR: Untranslated region
