# Supplementary figures and images for: MicroRNA involvement in mechanism of endogenous protection induced by fastigial nucleus stimulation based on deep sequencing and bioinformatics
Source: BMC Med Genomics. 2015 Nov 23;8:79. doi: 10.1186/s12920-015-0155-4 (PMC4657244; doi:10.1186/s12920-015-0155-4)

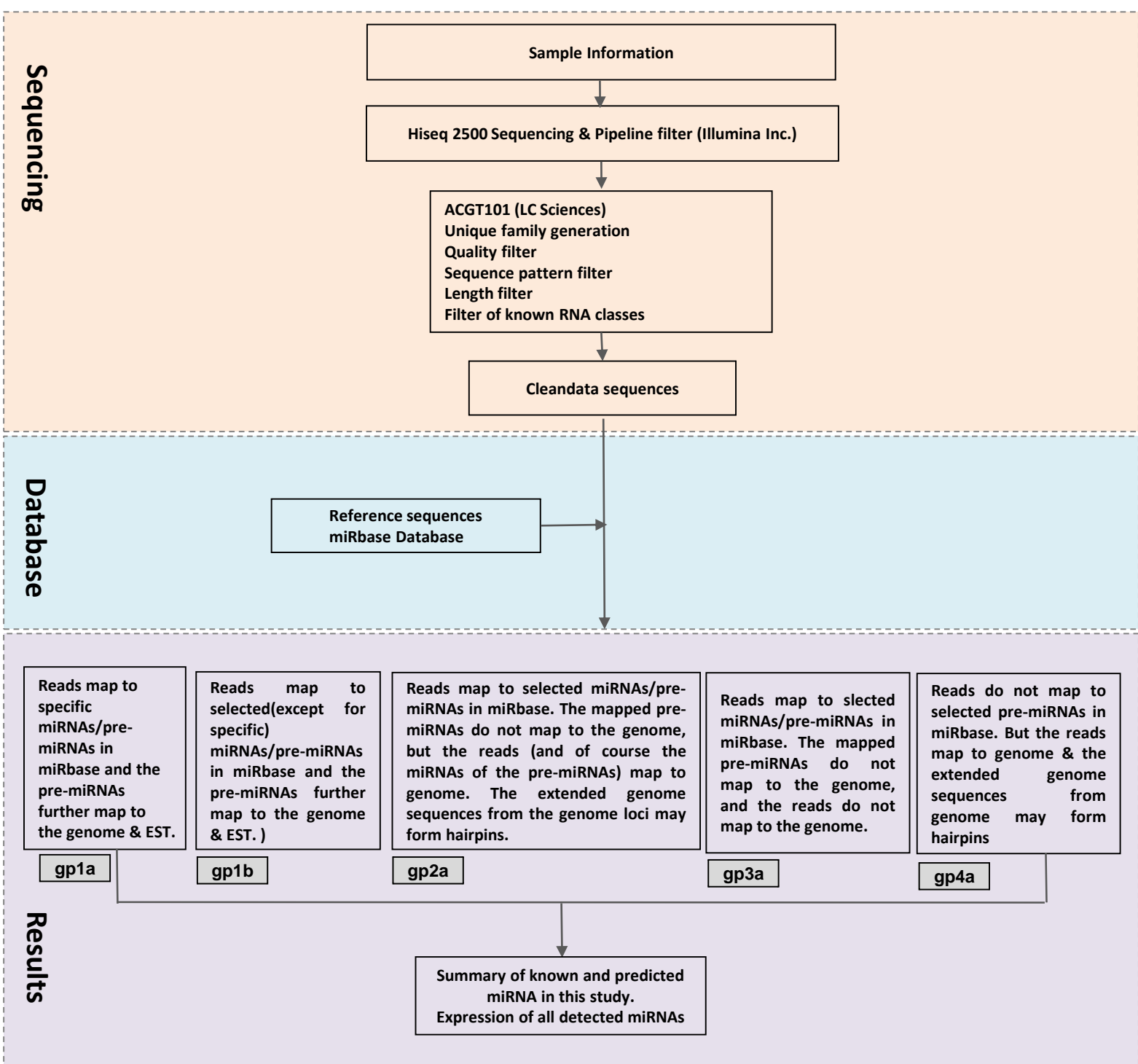

Figure S1. Analysis workflow of deep sequencing.

Supplement: Additional file 1: Figure S1. — Showed the analysis workflow of the deep sequencing. (PDF 97 kb) [file 12920_2015_155_MOESM1_ESM.pdf]
