# Supplementary material for: MicroRNA involvement in mechanism of endogenous protection induced by fastigial nucleus stimulation based on deep sequencing and bioinformatics
Source: BMC Med Genomics. 2015 Nov 23;8:79. doi: 10.1186/s12920-015-0155-4 (PMC4657244; doi:10.1186/s12920-015-0155-4)
Supplement: Additional file 2: Table S1. — Showed the enriched GO terms and KEGG pathway of predicted targets of top 20 abundant miRNAs. (PDF 15 kb) [file 12920_2015_155_MOESM2_ESM.pdf]

Table S1. The enriched GO terms and KEGG pathway of predicted targets of top 20 abundant miRNAs.

GO terms			KEGG pathway
**biological processes**	**cellular components**	**molecular functions**	
regulation of transcription, DNA-dependent	cytoplasm	metal ion binding	Metabolic pathways
positive regulation of transcription from RNA polymerase II promoter	nucleus	nucleotide binding	Pathways in cancer
signal transduction	integral to membrane	zinc ion binding	MAPK signaling pathway
positive regulation of transcription, DNA-dependent	membrane	protein binding	Neuroactive ligand-receptor interaction
ion transport	plasma membrane	ATP binding	Endocytosis
multicellular organismal development	nucleolus	DNA binding	Focal adhesion
response to drug	mitochondrion	receptor activity	Calcium signaling pathway
negative regulation of transcription from RNA polymerase II promoter	intracellular	sequence-specific DNA binding transcription factor activity	Regulation of actin cytoskeleton
transmembrane transport	Golgi apparatus	hydrolase activity	Chemokine signaling pathway
protein phosphorylation	extracellular region	nucleic acid binding	Cytokine-cytokine receptor interaction
proteolysis	cytosol	binding	Purine metabolism
positive regulation of cell proliferation	endoplasmic reticulum	protein homodimerization activity	Wnt signaling pathway
cell differentiation	extracellular space	sequence-specific DNA binding	Neurotrophin signaling pathway
negative regulation of apoptotic process	endoplasmic reticulum membrane	signal transducer activity	Insulin signaling pathway
metabolic process	membrane fraction	calcium ion binding	Alzheimer's disease
negative regulation of cell proliferation	cytoskeleton	protein serine/threonine kinase activity	Ubiquitin mediated proteolysis
cell cycle	protein complex	transferase activity	Tight junction
protein transport	perinuclear region of cytoplasm	RNA binding	T cell receptor signaling pathway
cell adhesion	soluble fraction	GTP binding	Axon guidance
apoptotic process	cell surface	peptidase activity	Protein processing in endoplasmic reticulum
