# Supplementary material for: MicroRNA involvement in mechanism of endogenous protection induced by fastigial nucleus stimulation based on deep sequencing and bioinformatics
Source: BMC Med Genomics. 2015 Nov 23;8:79. doi: 10.1186/s12920-015-0155-4 (PMC4657244; doi:10.1186/s12920-015-0155-4)
Supplement: Additional file 3: Table S2. — Showed the differentially expression pattern after 1-h of FNS. (PDF 321 kb) [file 12920_2015_155_MOESM3_ESM.pdf]

Table S2. The miRNAs altered after FNS

	3 h		6 h		12 h		24 h		72 h	
	log2(G2/G1)	p-value	log2(G2/G1)	p-value	log2(G2/G1)	p-value	log2(G2/G1)	p-value	log2(G2/G1)	p-value
rno-let-7a-5p	0.07	3.31E-01	0.23	1.76E-01	0.26	2.47E-02	0.00	9.64E-01	-0.13	6.65E-02
rno-let-7a-1-3p	0.94	1.57E-02	0.84	5.71E-02	2.36	9.53E-04	2.04	1.89E-04	-0.15	3.27E-01
rno-let-7b-5p	-0.05	3.03E-01	0.41	4.87E-02	0.40	9.20E-03	0.01	9.25E-01	-0.17	8.71E-02
rno-let-7b-3p	0.95	2.47E-02	1.09	4.08E-02	0.97	1.37E-02	0.37	1.03E-01	0.73	1.79E-02
rno-let-7c-5p	-0.09	1.68E-01	0.30	9.18E-02	0.23	2.64E-02	0.08	3.29E-01	-0.21	1.46E-01
rno-let-7d-5p	-0.03	1.50E-01	0.42	2.02E-02	0.30	6.71E-03	-0.11	6.44E-02	-0.37	5.65E-02
rno-let-7d-3p	0.86	2.72E-03	0.88	8.76E-04	0.68	6.09E-02	0.11	1.72E-01	0.16	3.57E-01
rno-let-7e-5p	0.13	1.55E-02	0.71	4.66E-03	0.33	3.80E-03	-0.45	1.14E-03	-0.55	2.65E-02
rno-let-7f-5p	0.12	4.18E-02	0.57	1.10E-02	0.31	2.23E-01	-0.35	1.41E-02	-0.56	2.11E-02
rno-let-7f-1-3p	-0.00	8.75E-01	-0.28	3.46E-01	1.25	1.91E-03	2.19	5.28E-03	-0.14	4.92E-01
rno-let-7f-2-3p	0.07	3.20E-02	0.42	9.05E-03	0.27	3.81E-02	0.23	5.45E-01	-0.24	1.03E-01
rno-miR-1-3p	0.78	1.49E-01	1.29	1.76E-02	3.57	4.95E-02	1.11	1.24E-01	1.23	1.18E-02
rno-miR-100-5p	-0.29	1.56E-03	-0.05	4.54E-01	-0.26	6.34E-02	-0.42	6.69E-02	0.05	6.70E-01
rno-miR-101a-3	-0.36	4.63E-02	-0.08	5.41E-01	-0.71	7.31E-03	-0.96	2.52E-04	-0.90	1.04E-03
rno-miR-101b-3	-0.69	1.94E-02	-0.28	4.91E-03	-0.80	6.63E-02	-0.81	6.56E-05	-0.93	2.52E-03
rno-miR-103-3p	-0.11	8.95E-01	-0.03	6.98E-01	0.45	1.96E-02	0.20	4.75E-02	-0.02	8.24E-01
rno-miR-106b-5	-0.68	1.24E-02	-0.75	1.43E-03	-0.87	1.95E-02	0.06	5.47E-01	-0.24	2.61E-01
rno-miR-107-3p	-0.14	1.45E-01	0.01	9.17E-01	0.39	2.14E-02	0.25	3.29E-02	-0.13	2.90E-01
rno-miR-1188-3	0.22	4.34E-01	-0.07	6.67E-01	-0.48	1.82E-01	0.54	1.46E-01	0.83	3.24E-02
rno-miR-1188-5	-0.92	2.25E-02	-1.98	3.07E-01	-7.20	4.63E-02	-2.63	1.30E-01	0.19	5.39E-01
rno-miR-122-3p	0.54	1.31E-01	-0.38	1.86E-01	0.90	6.69E-02	2.56	1.88E-03	1.48	1.33E-02
rno-miR-1224	1.00	5.93E-04	-0.76	2.52E-02	-1.60	2.81E-04	-0.23	2.11E-02	1.48	7.19E-06
rno-miR-124-3p	-0.14	8.72E-02	-0.23	1.13E-01	-0.21	1.45E-01	0.21	5.93E-02	-0.21	4.01E-02
rno-miR-1249	1.38	3.40E-02	1.55	3.72E-02	0.90	7.48E-02	1.81	3.83E-03	1.77	2.37E-03
rno-miR-125a-3	0.26	3.15E-01	0.58	4.16E-04	-0.44	2.47E-01	0.39	6.08E-02	0.80	1.56E-02
rno-miR-125a-5	-0.20	4.87E-02	-0.03	6.05E-01	-0.51	6.01E-03	0.15	1.59E-02	-0.64	7.55E-03
rno-miR-125b-2-	0.19	2.56E-01	-0.32	1.69E-01	-1.02	5.05E-02	-0.10	2.09E-01	-0.13	5.10E-01
rno-miR-125b-5	-0.10	1.50E-01	-0.36	2.92E-02	-0.65	4.94E-03	0.16	4.81E-02	-0.28	5.15E-02
rno-miR-126a-3	-0.34	2.29E-03	-0.21	7.39E-02	-0.47	5.33E-03	-0.53	3.25E-04	-0.67	1.42E-02
rno-miR-127-3p	-0.07	2.54E-01	-0.10	3.67E-01	-0.09	2.01E-01	0.21	2.57E-02	-0.24	1.03E-01
rno-miR-127-5p	-0.07	7.21E-01	-0.51	2.58E-02	-1.05	2.09E-01	-0.01	9.54E-01	-0.55	1.54E-01
rno-miR-128-3p	0.03	6.87E-01	-0.29	7.47E-02	-0.44	8.22E-03	0.16	2.00E-02	-0.41	5.04E-02
rno-miR-129-5p	0.21	1.34E-01	-0.01	9.49E-01	-0.74	3.20E-03	0.24	2.19E-02	0.09	2.96E-01
rno-miR-129-1-3	-0.98	9.06E-04	-1.39	2.82E-03	-2.53	4.91E-04	-1.29	3.14E-04	-1.21	2.25E-02
rno-miR-129-2-3	-0.72	1.16E-02	-0.74	1.24E-02	-1.23	2.55E-03	-0.78	4.42E-04	-0.81	2.66E-02
rno-miR-130a-3	-0.33	6.39E-02	-0.00	9.51E-01	-0.61	9.36E-03	0.00	9.76E-01	-0.28	8.47E-02
rno-miR-132-3p	-0.10	2.20E-01	-0.13	3.21E-01	-0.03	6.41E-01	-0.21	3.00E-02	-0.53	2.41E-02
rno-miR-132-5p	0.04	6.69E-01	0.17	2.39E-01	-0.18	1.92E-01	-1.15	4.99E-03	-0.99	6.27E-02
rno-miR-133a-3	-0.41	1.02E-01	-0.53	8.26E-02	-0.56	1.67E-01	-0.88	1.12E-02	0.00	9.13E-01
rno-miR-133b-3	-0.01	9.50E-01	-0.22	3.70E-01	-0.15	4.91E-01	-0.69	3.80E-02	0.47	1.73E-01
rno-miR-134-5p	-0.78	5.05E-03	-0.04	7.47E-01	0.22	7.54E-02	0.06	2.34E-01	-0.06	3.49E-01
rno-miR-137-3p	-0.78	9.75E-04	-0.40	7.63E-02	-1.81	8.37E-05	-0.74	1.52E-03	-0.35	2.43E-02
rno-miR-137-5p	-0.75	3.36E-02	0.06	5.86E-01	0.95	3.57E-04	0.17	3.83E-01	0.04	8.82E-01
rno-miR-138-5p	-0.55	5.61E-03	-0.46	8.29E-03	0.29	5.72E-02	0.30	9.25E-03	-0.13	2.01E-01
rno-miR-138-1-3	0.45	2.89E-02	0.42	2.65E-02	0.75	2.34E-02	0.65	4.30E-03	0.11	2.62E-01
rno-miR-138-2-3	-0.45	4.35E-03	-0.19	1.59E-01	0.76	3.36E-02	0.18	1.98E-01	-0.49	5.80E-03
rno-miR-139-3p	0.37	1.90E-01	0.83	1.31E-02	0.47	8.76E-02	-0.05	6.26E-01	-0.63	2.21E-03
rno-miR-139-5p	-0.01	7.54E-01	0.49	8.51E-04	1.11	3.39E-04	-0.16	6.21E-02	-0.89	2.46E-03
rno-miR-140-5p	-0.31	8.33E-03	0.08	1.41E-01	0.41	2.46E-03	0.16	1.64E-01	-0.10	1.84E-01
rno-miR-143-3p	-0.66	6.53E-03	-0.85	6.98E-04	-0.03	7.25E-01	0.09	2.44E-01	0.33	1.02E-03
rno-miR-145-5p	-0.62	1.55E-03	-0.70	1.01E-02	0.09	2.57E-01	0.03	7.39E-01	0.40	1.73E-03
rno-miR-146a-5	1.45	1.19E-03	0.63	2.38E-02	1.06	2.73E-02	-0.47	1.73E-02	0.10	4.31E-01
rno-miR-146b-5	0.88	3.42E-04	0.35	7.17E-03	0.28	5.03E-02	-0.32	3.87E-03	-0.44	2.55E-03
rno-miR-148b-3	-0.07	6.94E-01	0.49	3.99E-03	1.15	2.28E-01	-0.13	1.40E-01	-1.22	3.40E-02
rno-miR-150-5p	-0.22	2.87E-02	-0.07	1.83E-01	-0.44	7.54E-03	-0.47	6.06E-03	-0.83	9.90E-04
rno-miR-150-3p	-0.13	7.95E-01	0.10	7.01E-01	0.20	3.63E-01	0.11	6.53E-01	-1.24	2.05E-01
rno-miR-151-5p	-0.51	1.02E-02	-0.15	4.65E-02	0.42	9.81E-03	0.17	3.23E-02	0.06	7.09E-01
rno-miR-151-3p	-0.08	3.23E-01	-0.39	6.04E-03	0.14	2.04E-01	0.33	1.96E-02	-0.21	1.36E-01

	3 h		6 h		12 h		24 h		72 h	
	log2(G2/G1)	p-value	log2(G2/G1)	p-value	log2(G2/G1)	p-value	log2(G2/G1)	p-value	log2(G2/G1)	p-value
rno-miR-152-3p	0.48	3.77E-02	-0.19	5.34E-01	0.72	3.32E-01	0.12	3.62E-01	-1.59	3.13E-01
rno-miR-153-3p	-1.14	1.39E-04	-1.06	1.23E-03	-2.24	2.26E-03	-0.65	9.54E-03	-0.82	1.52E-01
rno-miR-154-5p	0.52	3.38E-03	0.15	8.18E-02	-0.39	1.46E-02	0.16	1.40E-02	-0.17	2.22E-01
rno-miR-15b-5p	0.45	1.05E-02	-0.13	2.69E-01	-0.63	4.37E-02	-0.50	1.37E-02	-0.61	4.61E-02
rno-miR-16-5p	-0.93	1.92E-04	-0.48	4.40E-04	-0.44	2.21E-03	-0.28	1.84E-03	-0.44	1.89E-02
rno-miR-17-5p	-0.33	8.57E-02	-0.38	3.47E-03	0.35	3.90E-02	-0.17	1.74E-03	-0.33	1.25E-01
rno-miR-181a-5p	-0.30	1.25E-02	-0.42	9.74E-03	-0.45	8.61E-04	0.14	2.64E-02	-0.10	3.05E-01
rno-miR-181a-2-	1.48	7.51E-03	-0.60	2.62E-01	0.57	4.10E-01	1.22	3.76E-03	2.16	4.32E-03
rno-miR-181b-5p	0.37	3.18E-02	0.53	1.16E-02	0.15	1.02E-01	-0.30	8.28E-04	-0.36	3.01E-02
rno-miR-181c-5p	1.45	5.71E-05	0.14	3.13E-01	-0.30	9.69E-02	0.08	3.24E-01	0.45	1.75E-02
rno-miR-181d-5p	1.68	9.76E-04	0.91	9.48E-04	-1.25	1.08E-02	-0.48	1.83E-03	-0.27	3.19E-02
rno-miR-181d-3p	1.37	2.67E-02	-0.09	9.38E-01	-0.85	3.12E-01	0.10	6.70E-01	-0.04	9.53E-01
rno-miR-182	1.42	2.86E-04	1.24	1.89E-02	1.26	1.23E-01	1.07	5.36E-03	-0.15	3.28E-01
rno-miR-185-5p	-0.06	2.32E-01	-0.20	1.05E-01	-0.03	3.08E-01	0.40	1.80E-03	-0.05	2.76E-01
rno-miR-186-5p	-0.18	3.94E-01	0.42	1.28E-01	0.96	7.47E-02	-0.43	1.62E-02	-0.72	2.62E-02
rno-miR-187-3p	-0.19	2.42E-02	-0.59	5.69E-03	-0.57	2.56E-02	0.36	1.48E-02	-1.46	2.22E-04
rno-miR-187-5p	1.55	9.91E-04	1.07	2.32E-02	0.68	3.36E-03	0.73	1.87E-02	0.86	8.16E-03
rno-miR-188-5p	0.95	1.28E-02	0.53	6.00E-02	0.39	3.58E-02	0.57	6.39E-02	1.31	5.09E-04
rno-miR-191a-5p	-0.25	2.95E-02	-0.15	1.85E-01	-0.32	1.88E-02	0.02	6.92E-01	0.00	9.90E-01
rno-miR-191a-3p	0.30	6.92E-01	5.74	5.11E-02	0.72	3.44E-01	0.72	1.29E-01	0.60	2.97E-01
rno-miR-192-5p	0.55	3.71E-02	0.51	1.38E-01	0.51	1.09E-02	-0.64	5.48E-02	-0.53	9.01E-03
rno-miR-193a-3p	0.61	1.16E-01	-0.41	4.71E-01	0.32	4.20E-01	-0.49	5.53E-02	-1.02	1.55E-01
rno-miR-194-5p	1.11	3.39E-02	0.25	3.13E-01	0.67	7.63E-02	-0.95	2.59E-02	-1.19	2.28E-01
rno-miR-194-3p	0.98	4.44E-03	0.30	3.58E-01	0.52	1.84E-01	-0.81	1.69E-01	-0.54	2.30E-01
rno-miR-195-5p	0.45	4.73E-03	0.20	9.75E-02	0.09	3.32E-01	-0.60	2.44E-03	-0.45	1.29E-02
rno-miR-195-3p	-0.04	8.81E-01	0.46	5.77E-02	0.11	6.97E-01	0.09	7.23E-01	0.08	9.94E-01
rno-miR-196b-3p	1.05	5.24E-03	-0.60	1.68E-01	0.76	3.19E-02	1.75	3.91E-05	1.77	1.06E-02
rno-miR-196c-3p	1.41	1.27E-03	0.48	1.13E-01	1.54	4.41E-04	1.38	3.64E-04	1.42	2.16E-02
rno-miR-199a-3p	0.84	7.82E-02	-1.13	2.12E-01	-0.53	1.43E-01	-1.31	4.10E-02	-0.97	2.98E-04
rno-miR-204-5p	1.26	5.79E-03	0.48	1.19E-02	-1.05	1.17E-02	-0.31	2.79E-02	-0.42	1.92E-02
rno-miR-204-3p	0.37	7.52E-02	-0.75	5.63E-03	-0.22	1.16E-01	0.37	1.67E-01	1.37	2.67E-03
rno-miR-206-3p	1.65	7.56E-04	1.71	1.83E-02	-0.96	8.37E-01	0.46	2.50E-02	-0.07	3.45E-01
rno-miR-208a-5p	1.54	2.36E-04	-0.50	1.03E-02	1.02	2.60E-03	-2.65	1.72E-02	-0.17	1.36E-01
rno-miR-20a-5p	0.10	5.89E-01	-0.53	1.54E-02	-0.52	2.91E-02	-0.46	4.19E-02	-0.36	7.34E-03
rno-miR-21-5p	1.84	2.69E-03	-0.32	2.50E-02	-1.63	1.33E-02	-1.46	1.53E-04	-0.70	6.91E-04
rno-miR-210-3p	-0.76	1.12E-02	-0.78	1.54E-02	0.27	4.74E-01	0.38	1.33E-01	0.33	3.45E-01
rno-miR-210-5p	1.89	3.61E-04	0.57	1.20E-01	1.45	5.56E-04	1.07	1.71E-03	1.28	2.79E-05
rno-miR-211-3p	1.80	8.11E-02	-0.04	9.74E-01	0.19	4.04E-01	0.59	1.04E-01	1.69	1.65E-03
rno-miR-212-3p	0.11	9.28E-02	-0.19	1.77E-01	0.55	1.04E-02	-0.39	1.76E-02	-0.54	1.24E-02
rno-miR-212-5p	-0.68	3.55E-04	-0.96	7.13E-04	-0.53	5.60E-02	-0.48	8.41E-03	-0.40	2.48E-02
rno-miR-214-3p	0.84	8.02E-03	-0.08	3.05E-01	0.89	1.72E-03	0.82	7.19E-04	2.09	2.24E-04
rno-miR-215	2.22	1.39E-03	1.56	2.44E-04	1.69	1.86E-02	0.16	3.45E-01	1.44	4.25E-04
rno-miR-218a-5p	0.21	4.03E-02	-0.15	2.41E-01	-1.53	1.54E-04	-0.61	2.34E-04	-0.81	4.28E-03
rno-miR-219a-2-	0.66	4.67E-03	0.45	6.45E-03	-0.39	2.40E-02	-0.17	1.72E-02	-0.14	2.28E-02
rno-miR-22-3p	-0.23	6.60E-02	-0.49	2.25E-03	-0.59	1.85E-04	-0.08	1.97E-01	-0.14	1.97E-02
rno-miR-22-5p	0.15	4.98E-01	0.49	4.92E-02	0.25	1.25E-01	-0.66	2.96E-03	-0.61	1.29E-02
rno-miR-221-3p	-0.08	2.22E-01	-0.06	5.03E-01	0.24	1.16E-02	-0.16	4.69E-02	-0.56	7.28E-04
rno-miR-221-5p	0.36	5.67E-02	-0.56	1.85E-01	0.17	6.59E-01	-0.77	1.56E-02	-2.35	5.29E-02
rno-miR-222-3p	-0.16	5.51E-02	-0.05	4.10E-01	0.31	2.18E-02	-0.03	6.19E-01	-0.61	8.19E-03
rno-miR-223-3p	0.66	1.07E-02	1.13	2.89E-02	1.21	1.53E-02	0.26	1.87E-01	-0.85	4.52E-02
rno-miR-23a-3p	-0.04	3.13E-01	0.04	7.56E-01	0.83	4.14E-03	-0.10	3.98E-01	-0.39	5.35E-02
rno-miR-23a-5p	0.06	9.14E-01	0.08	9.04E-01	1.25	4.78E-02	0.08	5.43E-01	0.90	2.15E-02
rno-miR-23b-3p	-0.05	3.71E-01	-0.02	7.98E-01	0.64	2.92E-03	-0.04	6.93E-01	-0.30	1.05E-02
rno-miR-24-3p	-0.41	2.48E-03	-0.38	2.31E-02	0.32	8.04E-03	0.20	1.53E-01	-0.18	4.07E-02
rno-miR-24-2-5p	0.79	6.50E-02	0.23	5.06E-01	0.67	2.64E-01	-0.26	1.71E-01	-0.35	3.67E-01
rno-miR-25-3p	-0.30	1.63E-02	-0.35	4.64E-03	-0.22	4.74E-02	-0.32	3.52E-02	-0.54	4.01E-02
rno-miR-25-5p	-0.14	5.48E-01	0.08	7.53E-01	0.06	7.87E-01	0.07	7.35E-01	-0.13	6.21E-01
rno-miR-26a-5p	0.05	3.99E-01	0.03	6.20E-01	0.18	7.87E-02	0.10	3.82E-01	-0.29	4.81E-02
rno-miR-26b-5p	1.25	1.08E-03	1.64	3.03E-04	-0.05	6.65E-01	-1.06	3.05E-06	-0.41	1.76E-02

	3 h		6 h		12 h		24 h		72 h	
	log2(G2/G1)	p-value	log2(G2/G1)	p-value	log2(G2/G1)	p-value	log2(G2/G1)	p-value	log2(G2/G1)	p-value
rno-miR-27a-3p	0.48	1.62E-02	0.22	7.20E-02	0.48	7.97E-03	-0.27	3.36E-02	-0.31	4.06E-02
rno-miR-27b-3p	-0.15	8.16E-02	0.05	5.56E-01	0.32	2.39E-03	-0.30	3.47E-02	-0.41	3.39E-02
rno-miR-28-5p	1.01	6.47E-02	0.05	7.35E-01	0.20	6.16E-01	-0.59	8.25E-03	-0.50	9.23E-02
rno-miR-290	1.59	2.71E-03	1.31	2.06E-02	0.57	8.15E-04	-0.70	7.73E-03	-0.42	6.57E-02
rno-miR-292-5p	1.07	1.04E-01	0.62	1.76E-01	-0.01	9.09E-01	0.46	1.03E-01	0.41	1.85E-01
rno-miR-294	0.28	4.72E-01	0.41	2.51E-01	0.48	9.53E-01	-0.02	8.76E-01	0.92	2.32E-01
rno-miR-295-5p	0.87	1.22E-01	0.52	8.69E-02	1.93	7.24E-03	0.98	8.95E-04	1.10	1.95E-02
rno-miR-296-3p	-0.29	5.79E-01	-0.76	6.55E-02	-0.69	4.13E-01	0.45	5.83E-02	0.98	1.17E-01
rno-miR-296-5p	0.87	9.22E-02	0.94	5.27E-02	1.16	1.82E-02	1.57	3.49E-03	2.08	1.20E-04
rno-miR-298-5p	0.04	9.76E-01	0.64	4.36E-02	-0.23	5.45E-01	0.98	3.14E-02	1.55	1.33E-02
rno-miR-298-3p	1.29	5.88E-03	1.39	2.45E-03	1.32	2.02E-01	0.61	7.82E-02	0.50	1.32E-01
rno-miR-2985	0.75	4.22E-01	0.57	2.67E-01	2.08	1.15E-01	2.02	2.47E-03	2.42	1.04E-02
rno-miR-29a-3p	-0.38	5.08E-03	-0.81	1.15E-03	-0.73	5.80E-04	0.06	2.65E-01	-0.04	5.92E-01
rno-miR-29b-3p	0.39	4.35E-02	-0.88	8.10E-03	-3.00	3.37E-03	-0.48	3.95E-03	0.22	3.77E-03
rno-miR-29b-5p	0.84	4.44E-02	-0.06	7.89E-01	-1.01	1.99E-01	0.19	2.59E-02	-0.16	5.18E-01
rno-miR-29c-3p	0.27	2.61E-02	-1.02	1.51E-03	-3.00	1.17E-04	-1.08	2.21E-03	-0.25	4.97E-02
rno-miR-29c-5p	-0.60	1.42E-02	-0.81	6.08E-03	-1.16	2.23E-02	0.03	7.72E-01	-0.35	1.15E-01
rno-miR-300-3p	-0.79	1.07E-02	-0.45	1.35E-02	-0.15	2.87E-01	0.41	1.41E-02	-0.23	4.43E-02
rno-miR-30a-5p	0.11	2.01E-01	-0.18	9.88E-02	-0.72	2.32E-04	-0.17	1.66E-02	-0.11	1.99E-01
rno-miR-30a-3p	1.41	3.98E-03	0.56	7.43E-02	-1.07	3.29E-01	-0.48	2.81E-02	-2.47	3.82E-02
rno-miR-30b-5p	0.65	1.14E-02	-0.09	4.97E-01	-1.05	1.06E-03	-0.83	1.55E-04	-0.59	3.11E-03
rno-miR-30c-5p	-0.18	5.46E-02	-0.30	2.21E-02	-0.43	4.03E-03	-0.19	1.64E-02	-0.45	4.49E-03
rno-miR-30c-1-3	0.82	1.82E-01	-0.11	8.34E-01	-1.51	2.96E-01	-0.61	5.40E-02	1.41	2.11E-01
rno-miR-30c-2-3	0.29	1.88E-01	-0.23	3.07E-01	-1.48	3.40E-01	-0.17	3.99E-01	-0.41	4.38E-01
rno-miR-30d-5p	-0.74	6.52E-04	-0.55	3.87E-03	-0.63	2.97E-03	-0.68	7.39E-04	-0.48	3.53E-03
rno-miR-30e-5p	0.25	4.32E-02	-0.27	2.10E-03	-1.48	1.78E-02	-0.58	4.46E-03	-0.24	1.94E-02
rno-miR-30e-3p	1.06	1.12E-02	0.81	3.66E-02	0.33	7.54E-01	-0.90	2.37E-04	-0.74	8.31E-02
rno-miR-31a-5p	-1.04	1.70E-04	-0.33	8.01E-03	0.15	5.52E-02	0.01	7.64E-01	-0.38	6.03E-02
rno-miR-3120	1.26	4.56E-02	0.94	3.95E-01	0.79	2.03E-01	-0.07	7.50E-01	-0.07	7.81E-01
rno-miR-32-3p	0.85	2.20E-03	-0.93	1.01E-02	0.84	3.48E-03	2.08	4.35E-03	2.92	1.20E-04
rno-miR-320-3p	-0.23	3.62E-04	0.34	2.58E-01	0.60	1.32E-03	0.68	7.81E-04	0.20	9.65E-02
rno-miR-320-5p	0.83	1.30E-02	-0.32	2.96E-01	1.88	1.12E-02	2.83	6.71E-05	-0.56	1.43E-02
rno-miR-322-5p	0.52	4.25E-02	0.79	4.27E-02	2.10	5.51E-03	-0.64	2.65E-03	-0.59	3.64E-02
rno-miR-322-3p	0.45	3.56E-01	-0.22	5.25E-01	1.50	1.46E-02	-0.64	1.31E-02	-0.82	6.08E-02
rno-miR-323-3p	-0.37	3.95E-02	0.50	2.41E-02	0.38	3.12E-02	-0.81	3.04E-03	-1.25	3.47E-02
rno-miR-323-5p	0.02	9.93E-01	0.29	5.11E-01	0.10	6.64E-01	-1.77	4.49E-01	-0.31	3.34E-01
rno-miR-324-3p	-0.77	2.53E-03	-0.28	9.30E-02	0.33	4.16E-03	0.25	3.49E-02	-0.24	1.65E-01
rno-miR-324-5p	-0.59	1.61E-03	-0.10	7.71E-01	0.52	5.34E-03	0.39	4.50E-03	0.05	7.14E-01
rno-miR-325-3p	-0.76	2.34E-03	-0.31	5.04E-01	-0.22	3.35E-01	-1.32	4.14E-03	-1.27	6.11E-02
rno-miR-325-5p	-0.59	1.31E-02	-0.64	1.34E-02	0.35	7.67E-03	-0.33	3.45E-03	-0.59	1.18E-02
rno-miR-326-3p	-1.24	8.24E-04	-0.87	2.79E-02	-0.47	4.32E-02	0.89	3.96E-03	0.23	2.28E-01
rno-miR-326-5p	-0.76	7.15E-02	-0.74	2.70E-01	0.30	4.38E-01	0.32	4.55E-01	0.03	9.25E-01
rno-miR-327	0.06	7.18E-01	-0.42	2.55E-02	0.17	1.76E-01	0.78	4.17E-05	1.73	4.94E-03
rno-miR-328a-3p	-1.38	7.23E-05	-1.24	1.69E-03	-0.50	2.07E-03	1.14	7.65E-04	0.37	4.22E-02
rno-miR-328a-5p	3.38	1.96E-03	-1.93	5.50E-03	0.08	6.34E-01	-0.46	1.03E-02	1.95	1.68E-03
rno-miR-328b-3p	0.10	6.17E-01	-0.26	4.87E-01	-0.82	3.39E-02	0.54	2.47E-02	0.65	4.96E-02
rno-miR-329-3p	-0.29	1.47E-02	0.07	3.01E-01	-0.02	7.15E-01	0.07	3.61E-01	-0.30	1.20E-01
rno-miR-329-5p	-1.27	5.55E-03	-0.91	8.98E-05	0.96	3.14E-03	0.40	4.34E-03	-0.19	3.60E-01
rno-miR-330-3p	-0.94	7.71E-04	-0.36	2.72E-02	0.14	1.31E-01	0.59	5.23E-03	0.12	4.08E-01
rno-miR-331-3p	-0.42	1.10E-03	-0.37	2.87E-03	-0.48	7.17E-03	0.13	1.50E-01	-0.07	5.45E-01
rno-miR-335	1.45	1.27E-04	1.15	5.38E-03	-0.18	4.79E-01	-0.97	6.19E-05	0.31	3.39E-01
rno-miR-337-5p	-0.89	1.00E-02	-0.64	1.84E-02	0.44	3.01E-03	0.07	1.44E-01	-0.44	1.22E-01
rno-miR-338-3p	1.39	1.02E-02	-0.66	2.57E-02	-0.60	2.21E-01	-0.60	1.87E-02	0.46	1.37E-01
rno-miR-338-5p	0.85	7.73E-04	0.47	1.28E-02	-0.00	9.60E-01	-0.37	5.11E-03	-0.08	5.80E-01
rno-miR-339-3p	0.31	9.56E-02	0.42	2.43E-01	0.43	2.65E-01	0.97	3.38E-03	1.18	8.55E-03
rno-miR-340-5p	-0.07	9.02E-01	0.94	6.12E-03	3.56	1.70E-01	-0.77	7.57E-04	-1.01	2.57E-01
rno-miR-341	0.01	9.02E-01	-1.22	5.19E-02	-4.23	1.09E-01	-0.74	2.52E-02	0.22	3.45E-01
rno-miR-342-3p	-0.42	7.94E-04	0.18	1.15E-01	-0.52	2.12E-02	-0.42	5.96E-03	-0.47	1.78E-02
rno-miR-344a-3p	-1.22	1.90E-03	-0.34	1.99E-02	-0.04	3.77E-01	0.69	2.75E-03	-0.18	2.87E-01

	3 h		6 h		12 h		24 h		72 h	
	log2(G2/G1)	p-value	log2(G2/G1)	p-value	log2(G2/G1)	p-value	log2(G2/G1)	p-value	log2(G2/G1)	p-value
rno-miR-344b-1-	0.06	9.77E-01	0.32	1.13E-01	0.25	6.71E-01	-0.16	3.36E-01	-0.11	6.15E-01
rno-miR-344b-5p	-1.27	4.85E-03	-0.93	3.53E-03	1.15	2.43E-01	-0.12	1.73E-01	-1.10	5.53E-02
rno-miR-345-3p	0.38	4.43E-01	-0.92	5.10E-02	0.54	4.83E-01	0.27	3.19E-01	0.82	1.04E-01
rno-miR-345-5p	-0.60	2.45E-03	-0.71	1.32E-03	0.23	3.03E-01	0.34	2.73E-02	0.43	9.05E-02
rno-miR-346	-0.36	1.64E-02	-0.37	1.49E-03	0.67	2.88E-03	1.34	8.18E-04	1.33	2.27E-03
rno-miR-347	-0.31	8.35E-01	-0.64	2.93E-01	-2.26	1.63E-01	1.08	5.67E-02	1.43	1.66E-01
rno-miR-34a-5p	-0.90	7.81E-04	-0.77	2.71E-03	0.17	7.41E-02	0.61	2.39E-05	-0.34	1.41E-01
rno-miR-34b-3p	0.75	7.56E-02	0.07	5.92E-01	-1.34	3.64E-01	1.09	3.09E-03	0.96	4.69E-02
rno-miR-34c-5p	-1.19	1.63E-02	-0.74	4.69E-03	-2.62	3.37E-01	0.10	3.40E-01	-0.56	1.48E-01
rno-miR-34c-3p	1.07	1.04E-02	-0.03	9.86E-01	1.08	3.24E-03	1.24	1.15E-02	1.23	2.16E-04
rno-miR-350	0.33	1.10E-01	0.09	6.52E-01	-0.44	1.30E-01	0.24	3.15E-01	-1.11	6.89E-02
rno-miR-352	0.97	6.22E-04	1.64	1.66E-03	-0.36	4.79E-02	-0.39	3.51E-03	-0.53	1.98E-02
rno-miR-3541	0.41	1.62E-01	1.26	6.29E-02	1.54	1.32E-01	2.42	2.17E-03	1.92	3.39E-03
rno-miR-3547	0.70	5.68E-02	0.62	4.11E-02	-0.56	3.03E-01	1.72	6.56E-02	1.63	6.48E-03
rno-miR-3552	0.89	2.20E-03	0.95	8.32E-03	1.05	6.95E-03	0.54	3.06E-03	1.00	3.55E-03
rno-miR-3557-3p	-0.54	1.39E-01	1.87	2.86E-01	1.18	1.23E-01	-1.13	5.96E-03	-0.28	4.40E-01
rno-miR-3557-5p	-0.24	4.71E-01	0.84	1.57E-01	5.69	1.10E-01	1.38	2.82E-02	-1.75	1.50E-01
rno-miR-3562	0.40	3.34E-02	0.07	7.02E-01	-0.18	2.49E-02	-0.09	5.10E-01	0.87	7.61E-02
rno-miR-3563-3p	-0.12	9.41E-01	0.43	1.66E-01	0.64	1.40E-01	-1.18	6.39E-03	0.24	8.38E-01
rno-miR-3563-5p	-0.25	3.51E-01	-0.49	7.93E-02	0.07	6.36E-01	-0.33	3.29E-02	-1.03	2.84E-01
rno-miR-3564	0.81	1.91E-02	0.27	5.37E-02	1.00	5.36E-03	0.41	7.53E-03	1.54	4.65E-03
rno-miR-3568	-0.25	1.62E-01	-0.34	4.92E-02	1.05	3.50E-02	0.43	6.33E-02	1.02	5.05E-02
rno-miR-3569	-0.79	1.28E-01	-0.46	3.36E-01	-0.91	4.25E-01	0.40	1.70E-01	1.22	1.81E-02
rno-miR-3573-3p	0.34	2.09E-01	1.70	1.71E-02	1.14	8.61E-02	2.68	1.17E-01	1.94	4.08E-03
rno-miR-3574	1.83	2.91E-05	1.11	1.35E-02	2.34	1.21E-04	0.83	1.93E-04	0.97	6.55E-04
rno-miR-3582	0.25	4.71E-01	0.46	1.25E-01	0.15	6.17E-01	0.12	5.34E-01	1.04	4.34E-02
rno-miR-3584-3p	-0.07	8.32E-01	0.68	2.34E-01	-0.05	7.78E-01	0.52	3.04E-02	1.30	2.61E-02
rno-miR-3584-5p	1.70	7.24E-03	-0.21	3.01E-03	0.18	1.85E-01	0.11	3.51E-01	1.85	6.70E-05
rno-miR-3591	0.79	1.53E-02	0.50	8.22E-03	1.36	1.08E-02	2.12	1.47E-03	0.93	2.72E-02
rno-miR-3593-3p	0.91	3.89E-03	1.05	1.87E-02	0.73	8.66E-03	-0.61	3.28E-02	-0.63	7.30E-02
rno-miR-3594-3p	-0.79	2.10E-01	-0.71	1.11E-02	-1.58	2.62E-03	1.19	1.63E-02	1.36	9.02E-02
rno-miR-3594-5p	-0.18	3.96E-01	-0.47	4.40E-02	-0.87	1.87E-02	-0.08	4.51E-01	0.93	2.81E-02
rno-miR-3596a	-3.06	9.89E-01	-0.24	2.27E-01	-4.40	1.40E-01	0.54	2.97E-02	0.19	7.56E-01
rno-miR-3596b	0.48	4.38E-01	1.33	3.16E-02	-0.90	6.01E-02	0.54	3.14E-02	0.36	4.01E-01
rno-miR-361-5p	-0.09	2.67E-01	0.38	7.92E-03	-0.07	2.26E-01	0.17	2.04E-02	-0.01	8.23E-01
rno-miR-361-3p	-0.94	2.21E-03	-0.22	1.05E-01	-0.19	1.08E-01	0.31	6.71E-03	0.50	8.71E-02
rno-miR-365-5p	2.02	1.07E-04	-0.16	7.65E-02	1.03	3.61E-04	-1.94	1.57E-03	1.41	2.01E-04
rno-miR-369-3p	2.54	1.29E-02	2.50	2.20E-03	-0.88	3.87E-02	-1.65	9.54E-04	-0.35	3.46E-01
rno-miR-369-5p	-0.10	2.89E-01	0.69	1.36E-03	-1.34	6.27E-03	-0.95	1.12E-04	-1.02	1.01E-01
rno-miR-370-3p	-0.46	5.99E-01	0.59	2.57E-01	-1.33	1.32E-02	-0.43	1.49E-01	1.65	1.73E-01
rno-miR-374-5p	1.21	2.73E-03	2.78	3.98E-02	0.92	6.18E-02	-0.66	2.48E-03	-0.52	2.02E-01
rno-miR-375-3p	0.25	5.90E-01	0.81	2.54E-01	-1.05	3.84E-01	-0.42	2.74E-01	-0.53	3.49E-01
rno-miR-375-5p	0.02	9.28E-01	1.53	1.20E-01	0.21	5.29E-01	0.83	2.15E-02	0.81	1.01E-01
rno-miR-376a-3p	0.03	5.75E-01	0.53	1.30E-02	1.07	4.93E-03	1.12	2.06E-03	-0.30	2.38E-01
rno-miR-376b-3p	-0.07	5.45E-01	0.73	2.65E-03	0.48	3.41E-02	-0.62	5.87E-03	-0.43	4.62E-02
rno-miR-376b-5p	-0.61	4.21E-03	0.54	7.38E-04	0.64	5.93E-02	-0.57	1.12E-03	-1.03	9.85E-03
rno-miR-376c-3p	-0.15	6.03E-01	2.73	2.14E-01	2.68	4.66E-02	-0.98	1.78E-03	-0.98	4.08E-02
rno-miR-376c-5p	-0.12	9.54E-01	1.77	1.78E-01	1.23	1.45E-01	-0.31	1.48E-01	-0.20	5.58E-01
rno-miR-377-3p	-0.13	5.15E-01	-1.57	2.05E-02	-5.46	1.24E-01	-0.56	1.13E-02	0.54	6.29E-02
rno-miR-378a-3p	-0.64	1.55E-03	-0.36	2.41E-02	0.50	5.01E-03	0.20	1.09E-02	-0.39	2.07E-02
rno-miR-378a-5p	-0.96	1.13E-01	-1.09	3.37E-02	-0.43	2.90E-01	0.44	2.45E-01	-1.00	1.29E-01
rno-miR-379-5p	-0.19	5.62E-03	-0.26	5.18E-01	0.13	4.83E-02	0.13	4.27E-03	0.09	3.55E-01
rno-miR-379-3p	0.10	6.03E-01	1.54	2.81E-02	0.52	2.25E-01	-0.79	4.12E-03	-1.85	1.81E-01
rno-miR-380-5p	-0.47	7.69E-02	-0.10	7.31E-01	-0.87	4.02E-01	-0.80	2.28E-02	-1.56	3.24E-01
rno-miR-380-3p	-0.50	3.49E-02	0.72	2.42E-02	0.68	8.59E-02	-0.74	1.88E-02	-1.14	7.74E-04
rno-miR-381-3p	-0.28	3.21E-01	0.62	8.84E-02	2.00	3.07E-01	0.15	1.34E-01	-0.32	3.26E-01
rno-miR-381-5p	-2.13	2.09E-02	-2.76	6.35E-02	-0.36	4.75E-01	0.20	5.83E-01	0.63	5.63E-01
rno-miR-382-5p	0.26	1.72E-02	0.36	1.98E-02	-0.17	7.50E-02	-0.07	1.24E-01	-0.17	8.03E-02
rno-miR-382-3p	0.48	3.75E-02	0.56	8.15E-02	1.74	2.59E-02	1.42	5.34E-04	0.46	1.26E-02

	3 h		6 h		12 h		24 h		72 h	
	log2(G2/G1)	p-value	log2(G2/G1)	p-value	log2(G2/G1)	p-value	log2(G2/G1)	p-value	log2(G2/G1)	p-value
rno-miR-383-5p	-0.45	1.47E-02	-0.42	7.95E-03	-0.24	1.80E-02	0.39	6.41E-03	-0.08	4.51E-01
rno-miR-384-3p	0.93	3.33E-03	0.69	1.72E-02	-1.30	4.07E-02	-0.94	3.67E-03	-0.43	4.47E-02
rno-miR-384-5p	0.71	8.64E-03	1.17	3.68E-06	0.67	8.08E-03	-0.30	1.94E-03	-0.74	9.91E-03
rno-miR-409-3p	-0.28	5.44E-03	-0.10	3.67E-01	0.24	1.22E-01	0.33	8.08E-04	0.19	3.36E-01
rno-miR-409-5p	-1.19	1.79E-01	-0.46	5.24E-02	-1.05	3.85E-01	-0.48	1.63E-01	-1.17	2.05E-01
rno-miR-410-3p	-1.02	1.07E-03	0.27	3.97E-02	-0.50	9.74E-02	-0.70	9.79E-04	-1.28	1.19E-02
rno-miR-411-5p	-0.99	1.00E-02	-0.96	2.05E-03	-1.52	5.67E-02	-0.66	5.98E-02	-0.73	4.04E-02
rno-miR-411-3p	-0.10	5.21E-01	0.99	6.46E-03	0.44	3.40E-01	-0.40	4.14E-02	-1.20	2.57E-03
rno-miR-412-5p	-0.45	4.24E-01	-0.30	1.82E-01	-1.19	3.72E-01	-0.38	3.00E-01	-1.09	1.86E-01
rno-miR-423-3p	0.09	6.60E-01	0.14	6.26E-01	-0.79	3.80E-01	0.58	3.73E-02	1.05	4.72E-02
rno-miR-423-5p	-0.20	6.03E-02	-0.79	1.38E-02	-0.30	4.67E-02	1.34	9.09E-05	1.85	3.61E-03
rno-miR-425-5p	-1.53	4.47E-03	-1.30	9.47E-05	-1.08	8.95E-03	-0.05	6.71E-01	-0.18	1.57E-01
rno-miR-431	-1.83	4.73E-02	-0.78	2.73E-03	-0.72	7.95E-02	-0.03	7.72E-01	-1.80	9.70E-02
rno-miR-433-3p	-0.58	2.20E-02	0.10	2.34E-01	0.13	7.84E-02	0.52	1.14E-02	-0.13	1.64E-01
rno-miR-434-3p	-0.36	1.89E-02	0.09	1.35E-01	-0.21	5.15E-02	-0.11	5.31E-02	-0.47	2.48E-02
rno-miR-434-5p	-1.51	2.60E-03	-0.78	3.03E-03	-0.81	1.41E-02	-0.09	3.87E-01	-0.45	2.38E-01
rno-miR-448-5p	-0.43	5.46E-02	-0.23	2.69E-01	-0.46	2.14E-01	-0.13	1.28E-01	0.05	9.24E-01
rno-miR-451-5p	0.11	5.64E-01	-2.10	8.46E-05	-2.03	2.02E-03	0.20	9.55E-02	1.87	2.18E-02
rno-miR-455-3p	-0.38	1.06E-02	-0.42	1.28E-02	-0.50	3.49E-03	0.03	7.45E-01	-0.37	1.23E-01
rno-miR-465-5p	-0.58	1.69E-01	-0.14	3.69E-01	1.53	2.55E-02	0.93	1.88E-02	1.33	6.29E-03
rno-miR-466b-5p	0.68	5.33E-02	-1.58	8.68E-05	-1.34	2.51E-04	2.49	3.09E-05	3.50	2.35E-05
rno-miR-466b-3p	0.25	7.39E-02	-0.01	9.50E-01	-0.21	4.72E-02	0.61	6.90E-03	0.76	1.97E-04
rno-miR-466b-2-	0.34	3.71E-02	0.01	8.92E-01	0.06	2.50E-01	0.69	7.30E-03	0.74	5.05E-04
rno-miR-466c-3p	0.25	6.61E-02	0.03	6.75E-01	-0.16	9.57E-02	0.61	1.19E-02	0.72	4.71E-04
rno-miR-466d	0.38	4.39E-01	-1.45	2.31E-02	-1.90	6.47E-02	2.56	4.34E-04	3.33	1.57E-02
rno-miR-483-5p	0.43	9.77E-03	-0.05	4.48E-01	0.07	4.59E-01	0.70	7.65E-03	1.46	8.43E-05
rno-miR-484	0.22	4.84E-01	1.21	3.12E-03	0.13	5.70E-01	1.59	9.66E-04	1.27	1.16E-02
rno-miR-485-5p	-1.37	1.53E-02	-0.20	9.49E-02	-0.19	1.71E-01	0.54	1.30E-02	-0.49	5.40E-02
rno-miR-485-3p	-0.01	8.94E-01	0.25	2.14E-02	0.43	7.34E-02	1.09	3.50E-03	0.70	4.60E-03
rno-miR-487b-3p	-0.42	3.03E-03	0.11	1.63E-01	0.52	1.93E-04	-0.15	6.01E-02	-0.07	4.34E-01
rno-miR-487b-5p	0.79	2.11E-01	1.95	2.91E-01	-0.69	7.11E-02	0.46	5.79E-02	-0.22	5.08E-01
rno-miR-489-3p	0.50	1.39E-01	1.20	1.88E-02	1.12	3.44E-02	-0.61	5.45E-02	0.18	4.28E-01
rno-miR-490-5p	0.18	4.54E-01	0.48	2.12E-01	1.62	1.69E-01	-0.74	2.11E-01	-0.53	1.15E-01
rno-miR-493-3p	-1.12	3.39E-01	0.50	3.58E-01	1.91	1.52E-01	0.89	1.28E-02	1.08	2.46E-02
rno-miR-494-3p	2.03	6.98E-04	0.59	1.16E-02	-1.12	4.57E-03	-0.72	4.64E-04	3.38	2.49E-04
rno-miR-494-5p	0.19	5.38E-01	0.51	1.58E-01	1.78	5.10E-03	1.87	1.44E-04	0.24	4.73E-01
rno-miR-495	-0.36	2.72E-02	0.79	1.51E-01	0.18	1.24E-01	-0.46	2.23E-02	-0.58	5.35E-03
rno-miR-496-3p	-0.17	8.41E-02	-0.15	9.79E-02	-0.77	2.43E-02	-0.70	5.44E-03	-0.94	7.21E-03
rno-miR-496-5p	-0.14	5.09E-01	-0.27	3.64E-01	1.10	2.59E-02	1.14	1.36E-02	1.00	2.56E-02
rno-miR-497-5p	-0.58	6.44E-02	-1.06	2.93E-03	-0.25	1.11E-01	0.40	7.15E-02	0.28	9.80E-02
rno-miR-500-3p	0.14	6.33E-01	0.02	7.06E-01	-0.36	2.53E-01	-0.06	7.21E-01	-0.74	1.73E-01
rno-miR-503-3p	-0.63	1.22E-01	-0.90	1.47E-02	-0.22	4.87E-01	-0.19	5.98E-01	0.13	8.21E-01
rno-miR-504	-0.74	5.07E-02	-0.87	1.68E-03	-0.58	6.07E-02	-0.01	9.13E-01	-0.13	5.91E-01
rno-miR-505-3p	0.57	1.99E-03	0.52	7.92E-02	-0.22	3.60E-01	-0.55	6.26E-02	-0.83	5.76E-02
rno-miR-505-5p	-0.88	1.40E-01	-0.41	8.29E-02	0.15	5.54E-01	-0.45	1.30E-01	0.06	8.13E-01
rno-miR-532-3p	-0.31	5.58E-01	-1.38	3.42E-01	-6.56	5.96E-02	0.60	5.95E-02	0.64	1.53E-01
rno-miR-539-5p	0.40	4.05E-04	0.91	4.85E-05	-0.00	9.86E-01	-0.29	1.49E-03	-0.17	9.33E-02
rno-miR-539-3p	0.44	1.34E-02	0.61	7.69E-02	1.13	8.66E-02	-0.66	1.03E-02	-1.17	8.91E-03
rno-miR-541-5p	0.69	4.71E-03	0.60	1.00E-02	0.63	1.44E-01	-0.26	2.46E-03	-0.48	6.49E-02
rno-miR-543-5p	-1.27	7.68E-03	-0.60	2.65E-02	-1.45	6.02E-02	-0.39	7.88E-02	-1.88	1.78E-03
rno-miR-544-3p	-0.18	7.26E-02	-0.22	4.43E-02	-1.35	8.29E-04	-0.34	9.94E-03	-1.09	1.23E-03
rno-miR-568	1.35	1.14E-03	1.44	1.72E-02	1.67	7.36E-03	0.45	1.30E-04	0.07	2.97E-01
rno-miR-598-3p	0.10	3.41E-01	0.63	1.33E-04	-0.55	1.37E-02	-0.00	9.44E-01	-0.46	5.26E-03
rno-miR-628	0.68	3.86E-01	1.09	4.16E-02	2.85	3.27E-03	1.45	2.16E-02	0.38	2.03E-01
rno-miR-652-3p	-0.27	1.03E-01	0.04	4.01E-01	-1.12	1.37E-02	-0.11	1.35E-01	-0.74	6.80E-03
rno-miR-652-5p	0.75	1.17E-02	0.09	2.74E-01	-0.82	2.01E-03	-0.07	3.50E-01	0.90	1.77E-02
rno-miR-664-3p	0.31	2.00E-02	1.45	1.93E-02	-2.27	6.33E-02	-0.51	1.62E-02	-1.36	1.20E-02
rno-miR-664-1-5	-0.57	6.49E-02	0.06	9.26E-01	-3.48	1.31E-01	-0.76	5.12E-03	0.58	2.69E-03
rno-miR-664-2-5	-0.79	1.53E-02	0.57	1.44E-01	-1.45	1.12E-01	-0.65	1.21E-01	-0.19	5.65E-01

	3 h		6 h		12 h		24 h		72 h	
	log2(G2/G1)	p-value	log2(G2/G1)	p-value	log2(G2/G1)	p-value	log2(G2/G1)	p-value	log2(G2/G1)	p-value
rno-miR-665	-0.11	2.00E-01	-0.86	3.58E-04	-1.07	2.83E-03	0.35	2.71E-02	1.86	1.13E-03
rno-miR-667-3p	-0.57	2.78E-02	0.21	3.23E-01	-1.49	1.58E-02	0.10	5.22E-01	-0.50	1.30E-01
rno-miR-667-5p	0.24	3.02E-02	0.77	8.66E-03	-1.00	1.85E-02	-0.34	1.37E-01	0.56	7.77E-02
rno-miR-668	-1.01	1.06E-03	-0.09	8.35E-01	-0.65	1.63E-02	-0.21	2.50E-01	-0.24	4.54E-01
rno-miR-671	-0.69	8.73E-02	0.90	1.99E-01	-0.18	6.00E-01	0.44	9.67E-02	-0.36	6.47E-01
rno-miR-672-5p	1.84	1.09E-03	2.30	4.25E-05	-1.12	6.96E-04	0.80	7.09E-04	-0.05	5.85E-01
rno-miR-674-3p	-1.36	3.15E-03	-0.18	7.00E-02	-0.01	9.46E-01	-0.32	1.83E-02	-0.85	1.27E-02
rno-miR-674-5p	-0.26	1.85E-02	-0.33	3.08E-03	0.51	1.85E-02	0.12	5.40E-02	0.32	8.10E-02
rno-miR-678	1.00	2.37E-02	0.48	1.80E-01	-0.14	5.90E-01	-0.24	2.41E-01	0.85	3.70E-02
rno-miR-702-3p	0.75	3.48E-02	0.66	1.44E-01	0.64	6.00E-02	0.79	8.94E-03	1.02	6.25E-02
rno-miR-702-3p_	-3.27	2.26E-01	-4.85	9.39E-02	0.87	7.28E-02	0.73	6.51E-02	1.12	5.03E-04
rno-miR-702-5p	0.63	1.57E-01	0.06	7.22E-01	-0.04	8.07E-01	0.16	2.29E-02	1.13	1.71E-02
rno-miR-708-5p	-0.51	4.52E-03	-0.24	9.22E-03	0.65	5.59E-02	0.12	1.83E-01	-0.16	3.85E-01
rno-miR-711	-0.35	4.05E-01	-0.02	9.04E-01	0.16	9.73E-01	-0.08	8.31E-01	1.02	2.49E-02
rno-miR-758-3p	-0.67	1.27E-01	-0.12	5.84E-01	0.07	8.04E-01	-0.49	3.71E-02	-1.40	1.04E-01
rno-miR-758-5p	1.69	1.95E-04	1.30	5.82E-03	1.96	2.00E-05	1.07	2.48E-04	1.05	7.46E-04
rno-miR-760-3p	0.22	7.64E-02	-0.40	1.44E-01	0.26	2.15E-01	0.08	2.18E-01	0.31	1.97E-01
rno-miR-760-5p	-0.29	5.96E-01	-1.18	2.73E-02	-0.41	8.57E-02	0.15	5.64E-01	0.64	2.18E-01
rno-miR-761	1.10	3.38E-02	0.61	4.15E-01	3.39	1.77E-01	0.59	2.58E-02	0.75	2.88E-01
rno-miR-764-5p	-0.04	8.83E-01	-0.67	7.75E-02	0.63	3.36E-02	0.83	6.54E-03	0.97	1.49E-02
rno-miR-770-3p	-0.74	3.50E-04	-0.39	2.97E-02	-0.05	4.45E-01	-0.01	9.47E-01	-0.65	3.08E-02
rno-miR-7a-5p	0.63	3.50E-03	1.09	1.13E-03	-0.88	2.06E-03	-0.24	2.37E-02	0.11	3.27E-01
rno-miR-7a-1-3p	-0.93	5.17E-04	0.03	9.57E-01	1.44	1.49E-01	-1.47	8.27E-03	-0.20	2.02E-01
rno-miR-7a-2-3p	-1.18	2.37E-03	-0.12	4.70E-01	0.04	5.00E-01	0.37	5.15E-02	-0.50	9.09E-02
rno-miR-7b	1.46	8.71E-03	1.68	1.05E-02	-0.30	8.04E-02	-0.66	6.39E-02	0.26	4.66E-01
rno-miR-872-5p	0.14	4.20E-01	-0.40	1.36E-02	-0.79	2.16E-03	-0.77	6.25E-03	-0.78	1.73E-02
rno-miR-872-3p	0.21	6.60E-01	0.73	2.50E-01	1.89	3.12E-01	-0.94	6.71E-02	-1.11	1.68E-01
rno-miR-873-3p	-1.70	1.23E-02	-0.55	3.50E-02	0.19	6.33E-01	-0.37	2.32E-01	-1.21	9.79E-02
rno-miR-874-3p	0.89	2.51E-02	0.03	9.22E-01	0.83	1.92E-02	-1.20	1.68E-03	0.36	2.63E-02
rno-miR-877	0.15	5.26E-01	0.20	4.34E-01	-0.35	1.00E-01	0.27	1.05E-01	0.60	6.89E-02
rno-miR-9a-5p	0.26	1.45E-02	-0.13	1.93E-01	-0.91	9.61E-04	-0.25	1.46E-02	-0.39	3.22E-03
rno-miR-9a-3p	0.09	2.76E-01	0.43	1.17E-02	-0.32	1.04E-02	-0.24	2.03E-02	-0.35	4.90E-03
rno-miR-92a-3p	0.68	7.21E-03	0.53	1.24E-02	-0.74	1.96E-02	-0.18	7.70E-02	-0.95	2.01E-03
rno-miR-92b-3p	-0.84	1.38E-03	0.04	4.03E-01	-0.08	3.87E-01	0.91	2.48E-04	-0.06	4.61E-01
rno-miR-93-5p	-1.19	5.68E-04	-0.42	2.96E-03	-0.11	1.97E-01	0.10	4.73E-02	-0.36	1.87E-02
rno-miR-93-3p	-0.14	5.11E-01	0.05	8.84E-01	1.40	1.05E-02	2.24	6.66E-03	-0.94	8.22E-03
rno-miR-98-5p	1.72	7.75E-03	1.82	1.08E-04	-1.13	9.50E-04	-1.22	7.73E-04	-0.22	1.27E-02
rno-miR-99a-5p	-0.74	4.94E-04	-0.04	5.90E-01	-0.94	8.63E-05	-0.31	1.19E-02	-0.14	7.85E-02
rno-miR-99b-5p	-0.62	5.26E-04	-0.07	2.62E-01	-0.22	1.98E-02	0.18	4.48E-02	-0.12	1.08E-01
